# Deputy Surgeon-General Arthur Guy Elkington

**Published:** 1911-06-17

**Authors:** 


Deputy Surgeon-General Arthur Guy Elkington, late of the Brigade of Guards, who died recently at Farnborough Park, Hants, in his seventy-ninth year, received his first commission in the Army Medical Service in 1853, and served in the Crimean campaign, 1854-55. He was mentioned in despatches and received the British and Turkish medals, with the Medjidie of the Fifth Class. He retired in November 1887.

